# Progress of Surgery

**Published:** 1875-01

**Authors:** Jno. E. Owens

**Affiliations:** Lecturer on Surgery, Rush Medical College, Chicago


ARTICLE II.

### PROGRESS OF SURGERY.

BY JNO. E. OWENS, M.D.,

LECTURER ON SURGERY, RUSH MEDICAL COLLEGE, CHICAGO.

1. Bloodless Surgery. Report of Prof. ESMARCH'S Address. (*The Medical Press and Circular*, London, Oct, 21, 1874.)

2. Bloodless Surgery, by means of "The New Septic Cautery." (*Braithwaite's Retrospect*, July, 1874.)

3. Amyloid and Fatty Liver in Relation to Operations. By RICHARD BARWELL, F. R. C. S. (*London Lancet*, Oct., 1874.)

4. Surgical Treatment of Aneurisms. By Mr. T. HOLMES. (*The Practitioner*, London, Oct., 1874 ; *British Medical Journal*, Aug. 8, 1874.)

5. Incision versus Excision of the Knee in Children. By Dr. EDWARD LUND. (*The British Medical Journal*, *The Boston Medical and Surgical Journal*, Oct. 29, 1874.)

1. During a visit to London, Professor Esmarch, in an address delivered before the Clinical Society, took occasion to expound his method for the performance of bloodless operations. He prefaced his remarks by observing that many surgeons with whom he had conversed during his visit, were imperfectly acquainted with his method. Some, he found, used it, but not rightly; others did not consider the prevention of loss of blood important.

The object of the method is to secure good results in surgery, and the proof of its value is to be found in its influence on the mortality after operations, especially after amputations of limbs. Prof. Esmarch claims to have obtained striking results in the comparison of statistics of cases in his own practice prior to and after the introduction of his method. Its employment is by no means limited to operations on the limbs, but may be readily applied to other parts. In cases of excision of the shoulder-joint, a piece of elastic tubing may be passed around the arm-pit, and held over the shoulder by an assistant. Professor Esmarch illustrated this by the following case: A man between fifty and sixty years of age, suffering from a myxo-sarcomatous tumor of two years' growth, the size of an ostrich egg, in the axilla. Its removal was absolutely necessary, on account of the intense pain it occasioned. The tumor was wedged in between the scapula and the chest wall, firmly adherent to and moving with the former, but not with the humerus. The growth was thought to originate from the sheath of one of the cords of the brachial plexus; and, on account of the probability of recurrence, removal of the whole arm with the scapula was decided on. The arm having been bandaged with elastic webbing as far as the shoulder, a portion of the clavicle (outer two-thirds) was removed in order to ligature the sub-clavian, the pulsation of which could not be felt from the surface. Both artery and vein were ligated, the cords of the brachial plexus cut across, and the arm, together with the scapula, was removed by anterior and posterior flaps. The edges of the flaps were united by sutures, and under the use of carbolized oil dressing they speedily united. The report does not inform us how much, if any, blood was lost during the operation.

Prof. Esmarch explained to the Society that erectile tumors of the scalp in children can be readily removed without hemorrhage, by the introduction of needles, and the application of elastic tubing around the tumor. The method can likewise be made effectual in operations upon the male genital organs. The following case will serve to illustrate its use upon these parts:

An old man suffered from extensive epithelioma of the penis, involving the under surface of the penis and the scrotum, forming a cauliflower mass the size of the palm of the hand. Urine escaped from a fissure in the centre of the growth. The inguinal glands were much enlarged, and there was great emaciation. For the operation, an elastic tube was passed around the root of the penis, over the front of the thigh on each side, and crossed over the sacrum. The penis and anterior wall of the scrotum were now removed, together with the enlarged glands in the groin, with the integuments covering them; an incision in the median line having been made, the scrotal flaps were made to cover the exposed surface in the inguinal regions, and the urethra attached to the edges of the wound. Shortly after the operation death occurred from extensive cancerous deposit in the lungs.*

With regard to the results of the method, of three hundred cases in which it had been used, no evil had followed. In the longest operation, which lasted two hours and a quarter, and which was in a case of necrosis of both tibiæ, with resection of one knee in addition, no harm resulted from the prolonged absence of blood.

2. The objection to the employment of Mr. Henry Smith's method for the removal of hemorrhoids (clamp and actual cautery) is to be found in the great inconvenience of heating the cautery by the ordinary bed-room fire, the bars of the grate being in many instances too high to permit the insertion of the point of the instrument in the midst of a clear bright fire, whilst the handle rests on the hearth; the alternative being to thrust the iron into the fire with the handle protruding horizontally, in which case it becomes heated in the stem as well as in the button, causing the handle to be dangerously "warm" to the operator.

Messrs. Matthews, of Portugal street, London, have invented for Mr. Henry Smith "The New Septic Cautery," an ingenious arrangement, which entirely removes all these inconveniences. "The Septic Lamp" is on the principle of the Russian blow-pipe, by which methylated spirit is boiled in an upper reservoir, furnished with a safety-valve, and the spray of spirit vapor ignited by a little flame beneath the upper reservoir. The jet of flame is protected by copper sides, on which the irons are

* (The operation in the particular case cited we do not consider judicious. The age of the patient, the great enlargement of the inguinal glands, the evident cancerous cachexia, forebode a speedily fatal termination of such cases after operation.—O.)

placed, and made hot at the button only. The whole affair folds compactly, in a little japanned box, which is furnished with a tin compartment to hold the spirit.*

3. In a clinical lecture, Mr. Barwell alludes to fatty and amyloid enlargements of the liver in cases of prolonged suppuration, produced by caries, necrosis and disease of the joints. He further remarks that a patient who has suffered long from free suppuration, whatever be its origin, falls into a state of considerable cachexia; the body loses much of its fat; the skin becomes pale, of an opaque hue, often also dry, rough and inelastic. In spite of general emaciation, the abdomen, more especially of children, is generally tumid, yielding a loud hollow note over the greater part of its surface. On passing, however, to the right side, this loud note will be dulled over a certain distance below the hypochondrium according to the extent of enlargement—sometimes as low as the crista ilii. The surface of the gland (liver) is quite smooth, and the organ not altered in shape, save that its lower edge may appear rounder and thicker than usual.

There is, then, a uniform increase in size, which does

* (The handles of some of the cautery irons crack, and the irons become spoiled in consequence of being frequently subjected to intense heat. This matter of heating the irons becomes at times a serious inconvenience, and renders impracticable the use of the cautery and clamp—a method which, for the cure of hemorrhoids, has no superior. Occasionally the room in which one is obliged to operate, has neither grate nor stove. In such a case, I sometimes use an ordinary charcoal furnace, to which there are grave objections, as it should not be used in the patient's room, during the administration of an anæsthetic. It is really objectionable in any part of a house. Moreover, the preparation of a fire in the patient's room, for the purpose of heating the irons, engenders a horror which should be avoided.

Messrs. Bliss & Torrey have just imported for me Messrs. Matthews' "Septic Lamp," above described. It is fitted in a little bag, containing Mr. Henry Smith's set of clamps, forceps, scissors, cauteries, etc., for hemorrhoids. The lamp in the japanned box fits neatly in the lower part of the bag, and the case of instruments in an upper compartment. By means of the blow-pipe lamp the button of the cautery iron is made hot in "two minutes."—O.)

not affect, by pressure or obstruction, the postal circulation, since ascites is not present; and though it may diminish, it does not annihilate the secreting function of the gland, since the fæces are colored with bile, and there is no jaundice. This enlargement is due to the deposition in the constituents of the organ of oil globules in the case of fatty liver, or of a material called amylaceous, in the case of amyloid or lardaceous liver. The latter consists in the gradual infiltration of the liver with a material which appears to consist of homogeneous molecules—apparently a low form of protean, which hardens, thickens and whitens the organ, and, in some cases, destroys its function. The tissue thus affected is changed from its normal type of high organization to the lowest type of organic matter, which scarcely possesses life or function, which is hardly capable of undergoing change —even that last change of dead organism, putrefaction. Most writers on this subject consider the malady incurable.

Fat, in the shape of oil globules, is frequently present in the liver as the normal and temporary result of absorption from the intestine of oily ingesta.

The accumulation of such substance in the cells of the gland to a morbid extent may be due to a variety of causes. It is not unusual in wasting suppurative disease, as of the bones or joints. Since in healthy life fat is frequently present in, and as often removed from, the liver, it is plain that recovery from its morbid accumulation in that organ is a much more easy process, and a far more likely event, than restoration to the norm of a liver infiltrated with so immobile a material as that which constitutes the amyloid deposit. We are, therefore, often presented with problems which are of high importance and of extreme difficulty. The first concerns our power, or want of power, to discriminate during life between these two forms of liver enlargement; the second, our faith, or want of faith, in the power of nature to cure the secondary malady, if it be amylaceous, when its exciting cause, the source of suppuration, has been removed.

Concerning the former problem, it must be said, that our means of differential diagnosis are very imperfect, the greater hardness or softness of the liver being our chief guide. Fatty liver does not reach the size to which an amyloid organ may attain.

Concerning the second problem, Mr. Barwell, so far as the liver is concerned, does not concur in the opinion that amyloid disease once commenced is of necessity a progressively fatal malady. Although it may be impossible to diagnose with certainty during life between these two forms of enlarged liver, we have nevertheless a certain and sure guide to practice.

The liver, as well as every other organ, must be carefully examined in cases of prolonged suppuration. If, after careful investigation, the absence of any sign of amyloid disease save the enlargement of the liver be determined, any operation for the removal of suppuration is not only justifiable, but our imperative duty. Without operation the patient must die; after operation, however hard, large, and changed be the liver, there is a fair, indeed a considerable, chance of recovery.

4. In his recent lectures upon this subject, Mr. Holmes gives the following conclusions as to the treatment of popliteal aneurism:

(1.) Rapidly growing aneurisms, with a thin or imperfect sac, are best treated by immediate ligature, especially when caused by recent violence; and the success of compression is doubtful in aneurisms growing towards the knee-joint, and in all others which advance rapidly.

(2.) The Hunterian ligature has been about twice as successful in modern hospital practice in this country as the results of the accepted statistics show it to have been.

(3.) The results of the compression treatment in the same hospital have given as yet about the same average results as those of the ligature; but these results might be much improved by a more careful employment of the method.

(4.) Too long persistence in compression is to be deprecated as being likely to interfere with the success of the ligature.

(5.) Flexion is often successful when used so as not to distress the patient, and is worthy of trial in all cases in which it stops or materially checks the pulsation, but should not be long persisted in when it is not at once beneficial.

(6.) We have no evidence showing the utility of, or the need for, the less usual forms of treatment, such as galvanism, coagulating injections, manipulations, temporary ligature, or the introduction of foreign bodies.

5. At the last annual meeting of the British Medical Association, Dr. Lund argued that in a large proportion of cases under twelve to fifteen years of age, incision was a better operation than excision, in advanced inflammation of the knee joint. His method is to open the joint by an incision in its external side, and through this, by means of a curved cutting hooked knife, all adhesions were broken down, or cut through, in order to permit the bones to be replaced in a straight line. It was rarely possible to effect the latter immediately, but slight pressure, long continued, and extension, produce excellent results. It was, however, one essential condition of the operation, that it should be conducted entirely upon Mr. Lisler's system of antiseptic dressing.

